# Methionine Diet Evoked Hyperhomocysteinemia Causes Hippocampal Alterations, Metabolomics Plasma Changes and Behavioral Pattern in Wild Type Rats

**DOI:** 10.3390/ijms22094961

**Published:** 2021-05-07

**Authors:** Maria Kovalska, Eva Baranovicova, Dagmar Kalenska, Anna Tomascova, Marian Adamkov, Libusa Kovalska, Jan Lehotsky

**Affiliations:** 1 Department of Histology and Embryology, Jessenius Faculty of Medicine, Comenius University in Bratislava, 03601 Martin, Slovakia; maria.kovalska@uniba.sk (M.K.); marian.adamkov@uniba.sk (M.A.); 2 Department of Neuroscience, Biomedical Center Martin, Jessenius Faculty of Medicine, Comenius University in Bratislava, 03601 Martin, Slovakia; eva.baranovicova@uniba.sk (E.B.); anna.tomascova@uniba.sk (A.T.); 3 Department of Anatomy, Jessenius Faculty of Medicine, Comenius University in Bratislava, 03601 Martin, Slovakia; dagmar.kalenska@uniba.sk; 4 Clinic of Stomatology and Maxillofacial Surgery, Jessenius Faculty of Medicine, Comenius University in Bratislava, 03601 Martin, Slovakia; libusa.kovalska@uniba.sk; 5 Department of Medical Biochemistry, Jessenius Faculty of Medicine, Comenius University in Bratislava, 03601 Martin, Slovakia

**Keywords:** methionine diet, hyperhomocysteinemia, morris water maze, neurodegeneration, wild-type rats

## Abstract

L-methionine, an essential amino acid, plays a critical role in cell physiology. High intake and/or dysregulation in methionine (Met) metabolism results in accumulation of its intermediate(s) or breakdown products in plasma, including homocysteine (Hcy). High level of Hcy in plasma, hyperhomocysteinemia (hHcy), is considered to be an independent risk factor for cerebrovascular diseases, stroke and dementias. To evoke a mild hHcy in adult male Wistar rats we used an enriched Met diet at a dose of 2 g/kg of animal weight/day in duration of 4 weeks. The study contributes to the exploration of the impact of Met enriched diet inducing mild hHcy on nervous tissue by detecting the histo-morphological, metabolomic and behavioural alterations. We found an altered plasma metabolomic profile, modified spatial and learning memory acquisition as well as remarkable histo-morphological changes such as a decrease in neurons’ vitality, alterations in the morphology of neurons in the selective vulnerable hippocampal CA 1 area of animals treated with Met enriched diet. Results of these approaches suggest that the mild hHcy alters plasma metabolome and behavioural and histo-morphological patterns in rats, likely due to the potential Met induced changes in “methylation index” of hippocampal brain area, which eventually aggravates the noxious effect of high methionine intake.

## 1. Introduction

An occurrence of neurodegenerative diseases rises as life expectancy increases, concomitant with metabolic disturbances [1,2,3]. Human neurodegenerative diseases represent a large group of neurological disorders with heterogeneous clinical and pathological expressions, affecting specific subsets of neurons in the brain, characterized by the progressive dysfunction, loss of neuronal structure and function, eventually resulting in the neuronal cell death [1,4]. Although an ascending number of genetic factors that may affect the risk of neurodegenerative disorders are being identified, emerging findings suggest that dietary factors could also play major roles in determining neurodegeneration [4,5].

L-methionine (Met), the essential sulphur-containing amino acid in proteins, plays a critical role in cell physiology [6]. Normal plasma concentration of Met ranges from 13 to 45 μmol/L [7], and it is a result of Met metabolism, daily intake of sulphur amino acids (SAA) and endogenous protein degradation. Interestingly, the recommended daily intake of around 10 mg/kg could be doubled and is still considered safe. On the other hand, conditions linked to the liver diseases or prolonged high Met intake in the form of fish, beef, eggs, or nuts could lead to dysregulation of Met metabolism [5,6] and to the increased Met in the blood–hypermethioninemia (hMet). Hereditary based hMet is a consequence of dysregulation of at least 4 genes included in Met metabolism [8], and both these conditions may increase Met levels up to seventy times.

Evidence indicates that an excess of Met could be detrimental and might increase the risk of developing several diseases, such as type-2 diabetes mellitus [9], cardiovascular diseases, certain types of cancer [10] as well as alterations in the nervous tissue. Experimental studies have shown that Met may be extremely toxic to the brain by inducing oxidative stress, DNA damage, decreasing Na^+^, K^+^-ATPase activity, dendritic spine density, changes in Aβ oligomers, tau phosphorylation, Wnt signalling and synaptic remodelling [6,11,12,13]. In clinical studies, we see an exacerbation of psychopathological symptoms in schizophrenia [14], neurological dysfunction and cerebral oedema [15,16].

On the other hand, dietary restriction of Met has been reported to extend lifespan, reduce obesity and decrease oxidative damage to DNA in the heart, and increase endogenous hydrogen sulphide production in the liver and blood [17].

Interestingly, with respect to the hMet, disturbances in Met metabolism result in accumulation of its intermediate or breakdown products in plasma, mostly homocysteine (Hcy), as a part of Met-Hcy cycle. In addition, if dietary intake of Met is high, as in the Met loading test, the transsulfuration capacity is exceeded and Hcy is excreted from cells and blood concentration of total Hcy (tHcy) increases [1,6,9]. Over the years, different hypothesis focused on Hcy toxicity have been developed. However, despite the efforts, none of them does clearly explain the Hcy biotoxicity. The three main pathways of Hcy biotoxicity discussed in the literature are: (i) protein structure modifications known as homocysteinylation; (ii) oxidative stress induction; and (iii) excitotoxicity [18]. High level of Hcy in plasma-hyperhomocysteinemia (hHcy) has been found in many studies to be related to the development of neurodegenerative disorders [11,12,19,20]. Besides, clinical studies link high levels of another intermediate product in the Met cycle, (Met sulfoxide), in plasma with ageing and Alzheimer’s disease (AD) [21].

Recent papers suggest that hHcy caused by high Met diet leads to neuro-inflammation, cerebrovascular microhaemorrhages, memory decline [6,22,23], as well as autophagy, apoptosis and synaptic remodelling [12] in different experimental models. However, how the influence of mild hHcy, as an outcome of Met diet affects the wild-type rat brain needs to be elucidated.

In our previous works, we documented that administration of hHcy by subcutaneous application of Hcy, led in rats to the disintegration of neuronal tissue in the cerebral cortex as well as the hippocampus [2,3,24,25]. Moreover, Tóthová et al. [26] have shown that hHcy in the same rat model triggers remarkable epigenetic changes with the hyperacetylation of histones, presumably due to the hHcy initiated DNA hypomethylation in the highly sensitive brain areas [27]. Additionally, in the established hHcy model induced by high Met diet, we have found changes in the metabolic ratio as well as pathological swelling in the hippocampus [2].

This paper describes the alterations in the histo-morphological patterns of the rat hippocampus, metabolic changes in plasma, as well as the behavioural alterations in the established model of hHcy induced by the 2 g/kg Met enriched diet.

Modifications in the “one-carbon metabolism” due to the excess of Met, may exacerbate the toxic potential of Hcy and its metabolites and affects the “methylation index“ with the impact to the gene regulation [27,28]. In this follow-up study, we demonstrate here that Met enriched diet induces injurious process, manifested by neuropathological pattern in one of the most vulnerable brain areas in rats, *cornu ammonis* 1 (CA1) of the hippocampus concurrent with changes in plasma metabolome and animal behaviour.

## 2. Results

### 2.1. Level of Total Hcy in Plasma

Total plasma Hcy levels in animals with 28 days of Met enriched diet (Met-C) was significantly elevated and reached 12.45 ± 3.9 μmol/L (*n* = 5) when compared to the male control (C) Wistar rats (6.98 ± 0.57 μmol/L, *n* = 5). We did not find statistically significant changes in the weight between control (461.5 ± 13.7 g) and Met-C (443 ± 11.6 g) group at the end of Met diet treatment.

### 2.2. NMR (Nuclear Magnetic Resonance) Analysis of Plasma Metabolites

From 22 evaluated metabolites (lactate, glutamine, histidine, isoleucine, leucine, lysine, phenylalanine, threonine, tryptophan, tyrosine, valine, pyruvate, citrate, acetate, alanine, glucose, succinate, 3-hydroxybutyrate, 2-oxoisocaproate, 2-oxoisovalerate, 3-methyl-2-oxovalerate and lipoproteins fraction (composition please see [29,30]), plasma levels of eight metabolites: glucose, acetate, 3-hydroxybutyrate, phenylalanine, tryptophan, tyrosine, histidine and lipoproteins fraction were significantly changed in rats subjected to Met diet administration against controls. Changes observed in plasma levels of three metabolites: leucine, isoleucine and valine were approaching the borderline of significance with a *p* < 0.08. Details are summarized in Table 1.

### 2.3. Cresyl Violet Staining

**Figure 1:**
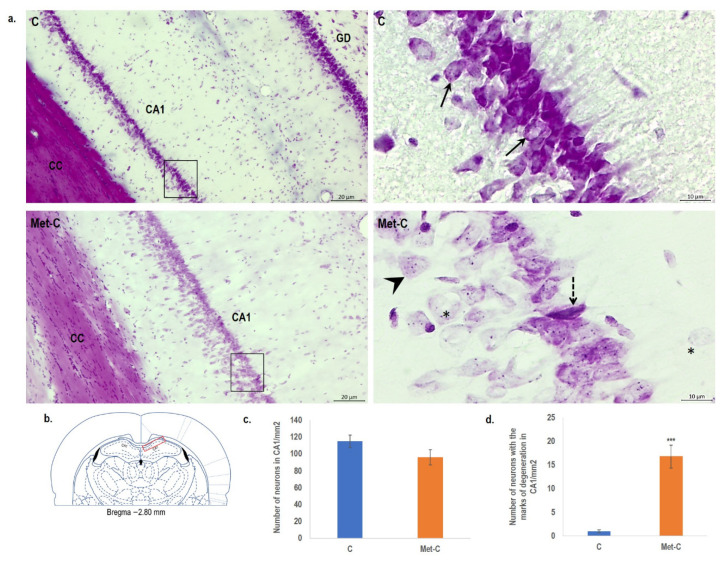
Cresyl violet stained rat brain sections and statistical evaluation of changes in the number of vital neurons in the CA1 region of the hippocampus of the rat brain. (**a**) Bright-field micrographs of CA1 region of hippocampus representing control (C) and Met-C group–low magnification in the left row, the right row represents high magnification (rectangle) of the corresponding group. Morphologically changed neurons are indicated by a dashed arrow (shrinkage of neurons) and by an asterisk (microvacuolisation and cell swelling), while arrows show vital neurons. CC–*corpus callosum*, GD–*gyrus dentatus.* Bar = 200 and 50 μm; *n* = 5/subgroup. (**b**) Schematic coronal rat brain section, redrawn according to Paxinos and Watson [31] representing hippocampus (blue rectangle) and CA1 area of the hippocampus (red rectangle). (**c**) The number of vital neuronal cells in the CA1 area of hippocampus in control and Met-C group. (**d**) The number of neurons with the marks of degeneration in the CA1 area of hippocampus in control and Met-C group. The significance of group mean differences was evaluated by unpaired *t*-test. Results are presented as mean ± SD, *n* = 5/subgroup, *** *p* < 0.001 versus the control group.

In order to assess the impact of Met diet enriched hHcy to the extent of neuronal damage, we used Cresyl violet staining commonly used to identify the neuronal structure in the brain which specifically stains Nissl bodies. In the control group (C), neural cells in the CA1 region appeared round with pale stained nuclei and purple Nissl bodies (Figure 1a).

On the other hand, the earliest neuronal alteration (lower intensity of staining, micro vacuolation of the cytoplasm) was detected in the Met-C group. Neurons in CA1 brain area showed morphological changes with signs of cell swelling (Figure 1a). More specifically, we observed considerable disintegration of proteosynthetic apparatus accompanied by cytoplasm shrinkage or microvacuolization. Despite neuropathological alterations, quantitative analysis of surviving neurons in the Met-C group displays no statistically significant changes (Figure 1c). However, after counting the number of neurons of the CA1 in the consecutive slides (Figure 1d) which manifested the marks of above-mentioned morphological changes, we found statistical significance in Met-C group of animals (16.8-fold increase versus control group; *p* < 0.001).

### 2.4. TUNEL (Terminal Transferase-Mediated dUTP Nick end Labeling) Assay

**Figure 2:**
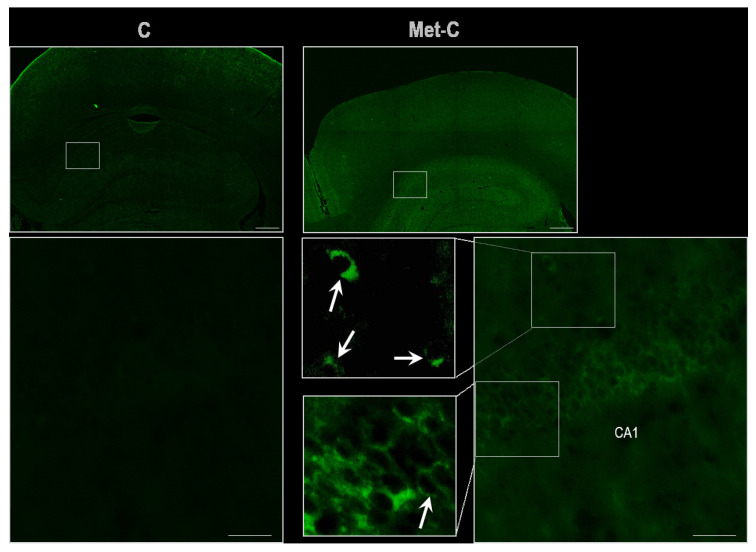
Demonstrative microphotographs of TUNEL staining of rat brain sections in the CA1 region of the hippocampus. Fluorescent micrographs of the hippocampus representing control and Met-C group–low magnification in the first line, the second line represents high magnification (rectangles) of the corresponding group. Arrows indicate perinuclear staining. Bar = 500 and 50 μm; *n* = 5/subgroup.

Almost no TUNEL positivity was detected in the control group in the CA1 region of the hippocampus. In Met diet treated animals, TUNEL positivity shares an unspecific pattern. Fluorescent signal was localized mainly in the perinuclear region with only faint nuclear positivity, likely due to proposed Met induced homocysteinylation of nuclear membrane proteins overreacting with fluorescein (Figure 2) [32,33].

### 2.5. Behavioural Tests

**Figure 3:**
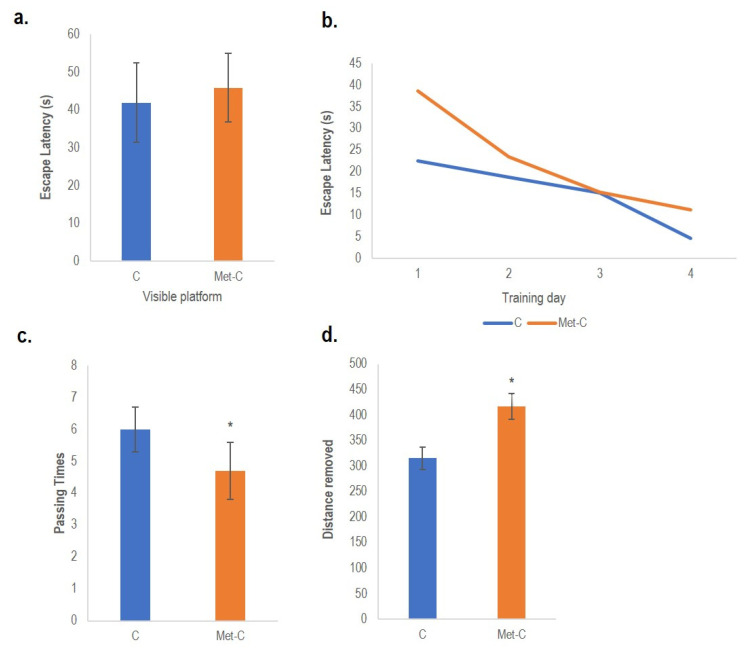
Spatial and working memory testing Path length (Distance removed) is shown as an index of spatial learning at the control and Met diet treated animals. Escape latency at the first day of training (**a**), escape latency during the whole training week on day 2–5. (**b**), passing times over the platform area (**c**) and path length spent in the target quadrant after removing the platform (**d**). Results are presented as mean ± SD for *n* = 5/subgroup. Behavioural analysis was performed by Student-Newman-Keuls test to compare the means of control (C) and Met-C; * p < 0.05 was considered to be statistically significant.

A spatial memory impairment analysis in rats induced by Met diet enriched hHcy was determined by the Morris Water Maze testing in control animals and animals treated with Met (Met-C) (Figure 3). Analysis of variance for repeated measurements indicated a day effect in the acquisitions phase of learning (a latency to find the platform).

On day 1 (visible platform trials), we have detected a modest increase in latency times in the Met-C-hHcy animals compared to the control groups (Figure 3a), indicating different motor and visual capabilities. This raise was not statistically significant. It seems that the ability of animals to see the flagged-platform and the cues in the surrounding environment in hHcy rats is negatively modified.

Later, on days 2–5 (Day 1 to 4 of hidden platform trials), we did not observe a considerable difference in the escape latency. As shown in Figure 3b, animals progressively improved performance over days as indicated by a decrease in time to find the immersed platform. There was just a slight difference between control and Met diet treated animals (hHcy) which suggests that both groups learned the paradigm to a quite similar degree.

On the 6th day, the probe trial was conducted with a removed platform. The trial lasted 60 s. The analysis showed a decrease in counts passing over the hidden platform in Met-C group, but this descent was not statistically significant. On the other hand, distance swam in the target quadrant was statistically significant in Met-C group. The path length increased in 32.2% (470 ± 25; *p <* 0.05) when compared to the controls (Figure 3c,d).

These findings suggest that in the model of hHcy induced by mild Met enriched diet rats manifest altered behavioural patterns with the negative influence on the spatial orientation and memory.

## 3. Discussion

Hereditary linked elevated levels of Met in infants can lead to intellectual disability and other neurological problems such as delays in motor skills, muscle weakness and liver problems [34]. Several studies have shown that high or chronic high intake of Met (in the form of proteins) could cause a loss of memory with Alzheimer’s-like pathological features in humans [6,35]. Studies in rodents shown that high Met promotes atherosclerotic plaques independently of Hcy levels [34] and in a similar study in Finnish men, Met showed the same effect [35].

Metabolic conversion of Met includes participation in the one-carbon metabolism and Met-Hcy cycle, which is one of the explanations of hHcy induction with the high Met diet [27]. A number of papers from other laboratories [12,20,23,36], but also the results of our previous experiments [1,2,24,25,26,37] demonstrated the pleiotropic neurotoxicity of hHcy.

Studies in humans shown that acute induction of transient hHcy by a supraphysiological oral load of Met, and, the studies on the hHcy experimental models [38,39], reported an impairment of the endothelium-dependent vascular function, in nitric oxide production/bioavailability initiation of Hcy-induced atherogenesis [40,41]. Moreover, evidence from clinical studies indicates that clinical hHcy is associated with vascular dementia, seizure, epilepsy and stroke with the common denominator–microhaemorrhages, which play a role in the acceleration of the development of brain atrophy in patients without dementia [12,42,43,44].

Outcomes from our previous works indicated that mild hHcy evoked by subcutaneous injection of Hcy in rats led to the exaggerated neurodegeneration and an altered pathomorphology in the hippocampus, entorhinal cortex as well as in more resistant cortical brain areas; parietal associated brain and primary motor cortex [3,24,37] with an accumulation of amyloid plaques around microvessels, as well as slightly elevated hyperphosphorylated tau positivity [25].

Established animal models are widely used to mimic dietary and/or metabolic disorders [45]. In the newly developed model of mild hMet linked with hHcy (evoked by 2 g/kg Met diet), we previously described oedema in the hippocampus with significant metabolic ratio changes, as well as neuropathology of this area [2].

Hippocampal pyramidal neurons of CA1 region are highly sensitive to insults such as ischemia, inflammation, hypoglycaemia or excitotoxicity when compared to CA3 and *gyrus gentatus* and is one of the most vulnerable brain areas has been studied for decades in the mechanisms of the onset and progression of neurodegeneration [46,47,48,49].

In our previous work, we found remarkable hippocampal histo-morphological changes such as oedema, alterations in number and morphology of astrocytes, neurons and their processes as detected by Fluro Jade C, NeuN, GFAP and β-tubulin staining [2]. Remarkebly, this paper in the same model documents an increased number of neurons with neurodegenerative pattern stained by Cresyl violet (for Nissl body) specifically in CA 1 region of rat hippocampus. This support the notion on potentially toxic effect of persistent high Met and consequent hHcy and is in line with our previous paper [2]. High Met diet induces also an alteration in hydrogen sulphide production, inflammatory factors and mitochondria function and morphology in male adult rats [50]. Notably, Met diet for 28 days was also associated with the induction of mild hHcy in wild-type adult rats, what was also shown in our previous works [2,51,52] and similar studies [22,53,54].

However, we have found no difference in the total neuronal counts of Met treated animals what correlates with the results of study of Zhang et al. [12]. Similarly, Singhal et al. [55] documented that high concentrations of Met and Met sulfoxide do not directly cause cell death but induce remarkable changes in astrocyte function and/or astrocytic end-foot disruption. Sudduth et al. [56] linked hHcy also with the vascular cognitive impairment, neuroinflammation, reduced synaptophysin and tau phosphorylation [57]. Combination of all mentioned events could finally, on the morphological level, affect astrocytes, as we have already described in our previous studies [2,24,25,52], or microglia and in turn neurons, detected in this study [58].

Additionally, experiments with gestational, neonatal or adults hMet with various duration on mice or rats show [12] an increased number of TUNEL+ cells with the increase in autophagosomes, apoptosis rates, DNA damage and caspase activity [59,60,61]. In our experimental design, TUNEL assay was not able to unequivocally detect statistically significant changes in the CA1 region, likely due to the duration of Met treatment (four weeks). However, study shows an unspecific TUNEL staining to the perinuclear region. This might be the outcome of potential hHcy- induced homocysteinylation and S-nitrosohomocysteine incorporation into proteins of nuclear membrane overreacting with fluorescein [32,33]. It seems from our experiments that duration of Met treatment might play a more decisive role to manifest the onset of apoptotic patterns in injured cells. On the other hand, morphological changes in neurons’ vitality, microvacuolisation or shrinkage of cells in the CA1 region seen in this study might finalize the pathology of cellular damage or delayed cell death, and by other studies using high or chronic Met diet treatment [6,12,62]. Remarkably, concurrent modification in the “methylation cycle” could lead to the DNA hypomethylation [26,27,63], followed by reduction of the regenerative potential of cells and other consequences such as dysfunction of the blood-brain barrier and onset of microhaemorrhages, all factors contributing to the cognitive decline of animals [12,23]. This also corresponds with outcomes of clinical studies [64,65] as well as results of animal behavioural analysis.

NMR metabolomic analysis of animal plasma showed alteration in energy metabolism in rats treated with 2 g Met diet. Decreased utilization of glucose is balanced with increased utilization of triacylglycerols (as the main component of lipoprotein fraction), to coordinate cellular function via epigenomic regulation [66] by its influence to the epigenetic histone acetylation [67] to coordinate, growth, apoptosis, and survival of glial cells and neurons [68]. Furthermore, relative levels of plasma amino acids: phenylalanine, tryptophan, tyrosine and histidine were significantly decreased in Met overfed rats. Additionally, boundary significant (*p* < 0.08) decrease was observed for plasma leucine, isoleucine and valine. All mentioned amino acids are essential what may suggest decreased protein intake in Met diet treated rats. As essential amino acids are basal building blocks of proteins, thus the observed changes may cause a decreased protein synthesis, which is in correlation with findings from histo-morphological analysis (Cresyl violet staining).

Cognitive, learning and memory performance depends on complex synaptic connections between sufficient neurons [12]. In fact, studies focusing on the effect of high Met diet induced hHcy on the behavioural changes are not always concordant. Several aspects can explain more or less contradictory results linked with Met treatment, such as: (i) different methodology, (ii) dose or duration of Met application, (iii) folate and B vitamin deficient diet, (iv) different species or strains of animals (iv) or by use of knockout animal models, (v) as well as the age of the treated animals. All stated conditions may have an impact on the brain Hcy levels and so the extent of Hcy neurotoxic effect reflected to the behavioural changes [6,19,23,42,62]. In this study, animals treated with Met manifested an impairment of spatial and learning memory, as well as an increased escape latency-the longest during the last training day and the higher swam distance. Remarkably, other papers documented a direct connection between the Met dose (Met ≥ 100 mg/kg) and diverse behavioural responses or abnormalities in the behavioural profile [6,66,69]. Recently, it was shown by Tchantchou et al. [23] that intraperitoneal 0.3 g/kg Met injection for 7 days in rats with induced brain trauma injury, to an increase in anxiety-like behaviour, alteration in the tight junction protein endothelial dysfunction, vascular microbleeds as factors of dementia. Moreover, the occurrence of Met metabolites was linked to memory deficit [70,71]. Clinic study in psychosis (FEP) [72] found Met/Hcy ratio, proposed as risk factor for developing psychosis.

Moreover, Met can undergo spontaneous self-assembly to form amyloidogenic aggregates [12], and an increased Met oxidation in the apolipoprotein A-I could nucleate amyloidogenesis, which eventually leads to the aggregation into amyloid fibrils [13,25]. Above mentioned studies correlate with our observations and suggest the neurotoxic effect of high Met to hippocampus which can eventually follow by cognitive decline in rats. Because memory impairment is frequently the earliest symptom of dementia [46,47,48,73,74], we hypothesise that Met induces neuropathological changes in the hippocampus link with the memory impairment at the very first stages of Met/Hcy neurotoxicity.

Taken together, our data show that model of mild hHcy as an outcome of 2 g/kg Met diet, remarkably affects the onset and possible progression of hippocampal neuropathology, and alters plasma metabolome as well as the behavioural pattern in rats. Our results can contribute to the valuable findings applicable in clinical studies and suggests the precaution of high protein diet (especially food with high Met content and low B vitamins) in the possible risk of human neuropathology.

## 4. Materials and Methods

### 4.1. Induction of Mild hHcy by Met Enriched Diet

Adult male Wistar rats (Velaz, Prague, Czech Republic) 5–6 months old and weighing 300–400 g (mean body weight of 335 g, total *n* = 20) were used. Animals were kept in air-conditioned rooms under the standard conditions, temperature (22 ± 2 °C) and 12 h day/night cycle. Food and water were available *ad libitum*.

Animals used in this study were treated in accordance with guideline for Animal Care and Health of the State Veterinary and Food Department of the Slovak Republic (approval number 727/12-221 for animal experiments). Experiment was implemented according to Directive 2010/63/EU for European Parliament and the Council on the Protection of Animals Used for Scientific Purposes.

All the conditions as well as the induction of mild hHcy were identical with our previous paper [2]. Rats were subjected to Met enriched diet throughout 28 days before the experiment. Met (L-methionine, Sigma-Aldrich, Taufkirchen, Germany) was given in drinking water at a dose of 2 g/kg of animal weight per day, according to Xu et al. [53]. All animals were weighted at the day 0, 3, 7, 14, 21 and 28. The daily volume of water intake was measured at 47.28 ± 5.91 mL for rats in both experimental groups. After this treatment, rats evoked moderate hHcy as we described in our previous papers [2,47,48]. At the 29th day, in control and animals treated with Met diet (10–12 h after the last Met treatment), peripheral blood samples (1.5 mL) were collected from retro-orbital venous plexus, immediately cooled on ice and centrifuged. The supernatant was collected and plasma was stored at –80 °C. According to the other experimental [6,11,12,19,22,53,56,57] and clinical [35,38,39,40] studies, for evaluation if the dietary induction of mild hHcy was successful, the total plasma Hcy was evaluated. The plasma Hcy levels were measured by commercially available enzymatic assay with Hcy Liquid Stable Reagent Kit (Axis-Shield Diagnostics, Scotland), according to the manufacturer’s instructions followed by analyses with an automatic biochemical analyser (Siemens ADVIA 1650).

Animals were divided into 2 groups according to the analysis which they were proceeding. The first group of animals underwent histological analyses (*n* = 5/subgroup). The second group of 10 animals was proceeded by behavioural analysis (*n* = 5/subgroup). After a particular period, the animals were sacrificed in mild sevoflurane anaesthesia in accordance with the ethical principles. Brains were rapidly dissected from the skull and processed for future procedures.

### 4.2. Experimental Groups of Animals

The rats were divided into following groups:

Group 1:

Subgroup 1: control animals (C, *n* = 5).

Subgroup 2: the animals after 28 days on Met diet (Met-C, *n* = 5).

Group 2:

The second group consisted of 10 animals (5 animals/subgroup) which underwent the same procedures as subgroups 1 and 2, but due to different handling and analysis methods, these were animals separated into independent group.

### 4.3. Behavioural Analysis

In order to evaluate the reference memory through a spatial search strategy, the Morris water maze test was performed at the day 29 of Met diet enriched hHcy. *Apparatus.* The circular open field water maze (Ugo Basile, IT) was built of blue fibre-glass with a diameter of 1.8 m and height of 60 cm. The tank was filled with tap water (25 ± 1 °C) to a depth of about 30 cm. The only possibility for the animals to escape was a clear Plexiglas platform (10 cm in diameter). This platform was immersed approximately 2 cm below the water surface. The tank was divided into four equal imaginary quadrants and four positions for starting (N: north, W: west, S: south, E: east). A circular platform was put into the water tank in the centre of NW quadrant (target quadrant). For making the water opaque we used non-fat dry milk. *Experimental procedure*. The rats underwent 3 training trials each day for 4 successive days. The rats were adventitiously placed into the water maze at starting positions facing the wall of the tank in each trial. The day before the trial begun, the rats were adapted for a stay in the pool without the platform for 1 min. The time and length of the path necessary to reach the platform was recorded. Once rats reached and climbed onto the platform, the trial was terminated and the animal stayed on the platform for 20 s. The limit for reaching the platform was set up to 2 min. If rats did not find the platform within this time, they were guided to it. A maximum of 60 s was assigned as latency. Thereafter, rats were returned to the home cage until being released for the next trial. The whole testing lasted for 6 days. For probe trials, the platform was removed and rats were released for free swim in a duration of 60 s. The time lapses of swim path crossing the platform area and path length were recorded. Swimming paths were monitored by an infrared camera connected to a tracking system. For each training trial, as well as for day 1, the swimming time to find the platform (escape latency) was recorded. At the end of the trials, the animals were dried using a clean towel and placed under a heating lamp in a holding cage before they were returned to their home cages [51].

### 4.4. NMR Analysis of Plasma

The whole procedure regarding blood sampling, plasma deproteinization, NMR measurement, data processing, evaluation and statistical analysis was carried out identically with our previous work [30].

### 4.5. Cresyl Violet Staining

After 28 days of Met diet (at the 29th day), control and Met-C groups (*n* = 5/group) were placed in an anaesthetic box and put to sleep by spontaneous inhalation of 3.5% sevoflurane in a mixture of oxygen and nitrous oxide (33/66%). Animals were subsequently transcardially perfused with 0.1 mol/L phosphate-buffered saline (PBS, pH 7.4) followed by 4% paraformaldehyde in 0.1 mol/L PBS (pH 7.4) [24]. After perfusion, all animals were decapitated. The brains were removed from the skull and submerged overnight in the same fixative at 4 °C. Lastly, the rat brains were placed in 30% sucrose for the next 24 h at 4 °C. The rat brains were embedded with embedding medium (Killik, Bio Optica, Milano, Italy) and promptly frozen by a fast-cooling boost in a cryobar Shannon Cryotome E (Thermo Scientific, Waltham, MA, USA) and cut in 30 μm thick coronal slices, using the stereotaxic coordinates of the rat brain atlas of Paxinos and Watson [31] as a reference. The coordinates (from Bregma) were approximately −2.80 mm to −3.80 mm. The sections were placed at Superfrost Plus glass (Thermo Scientific). Sections were afterwards dehydrated with ethanol in descending grades (100–70%; *v*/*v*), and then washed in distilled water. The sections were stained with 0.1% (*w*/*v*) cresyl violet and examined under the light microscope (Olympus BX41, Tokyo, Japan). The number of surviving neurons or neurons with marks of degeneration (shrink bodies or microvacuoles in the cytoplasm) in *cornu ammonis* 1 (CA1) of the hippocampus per 1 mm^2^ was counted as a neuronal density in the hippocampus.

### 4.6. In Situ Labelling of DNA Fragmenattion by TUNEL Assay

*In situ* nick end labelling of nuclear DNA fragmentation in the sections were performed in a humid chamber for 1 h in the dark at 37 °C with a TUNEL detection kit (In Situ Cell Death Detection Kit, Fluorescein, Roche) following the manufacturer’s instructions. For each experiment, a positive control was prepared by treating the sections with 1 U μL–1 DNase I for 10 min at 37 °C before labelling, as above. The negative controls were labelled in parallel, except for the absence of the enzyme terminal deoxynucleotidyl transferase (TdT). The labelling reaction (TUNEL) was stopped with 2 × SSC (300 mmol/L NaCl, 30 mmol/L sodium citrate) and then the slides were rinsed with PBS (pH 7.2). The slides were counterstained with Fluoromount™ Aqueous Mounting Medium (F4680, Sigma-Aldrich), for fluorescence microscopy and kept in the dark at 4 °C until a microscopic observation was conducted.

### 4.7. Quantitative Image Analysis

Using a cryostat, 30 μm-thick serial coronal sections were collected at every 3rd section (total 1000 μm, between −2.80 mm and −3.80 mm from bregma) and were further processed for staining.

**Figure 4:**
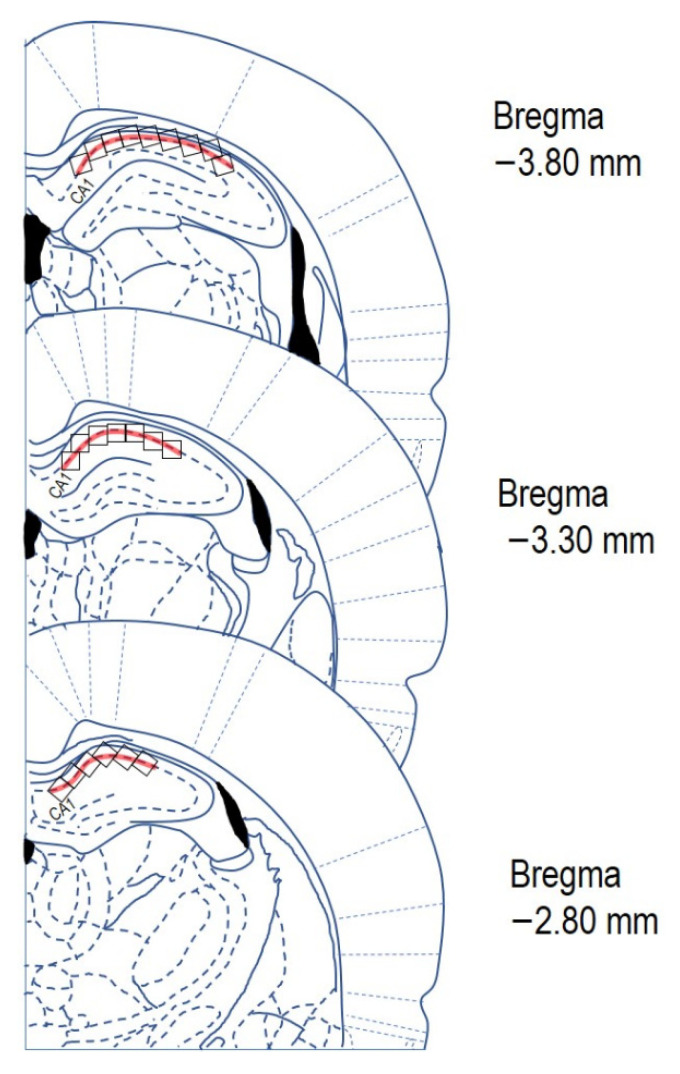
Schematic representation of three rat brain coronal sections (redrawn and modified according Paxinos and Watson [31]). Red line indicates the area of CA1 where the neurons have been counted. The counting area was divided into the counting grid 0.3 × 0.3 mm (black squares). Each section was counted in the CA1 at least in three of microscopic fields (black squares). Stereotaxic coordinates (from Bregma): −2.80 mm to −3.80 mm.

All quantitative analyses were performed in a double-blind manner by two skilled observers. The number of vital neurons, degenerative neurons (shrink bodies or microvacuoles in the cytoplasm; Figure 1), and TUNEL-positive neurons (Figure 2) was counted in the CA1 hippocampal region indicated in Figure 4 on three random microscopic fields (Figure 4).

Then, the results from 6 sections per animal were all added and multiplied by 3 (three different microscopic fields of each CA1) to estimate the total number of vital/degenerative or TUNEL-positive neurons.

The brightness and contrast of each image file were uniformly calibrated using Adobe Photoshop CS3 Extended, version 10.0 for Windows (Adobe Systems, San Jose, CA, USA). Images of the coronal brain sections from both experimental subgroups of the group 1 were exported in tiff format and evaluated using ImageJ software (NIH, Bethesda, MD, USA). First RGB channels were converted to 8-bit greyscale images. Threshold levels were adjusted from 13 pixels (min) to 255 pixels (max). Particle analysis was completed based upon size restrictions of 0 cm^2^-infinity leaving morphology unspecified. The counting parameters for Cresyl violet and TUNEL assay in 3 different areas of CA1 region of the rat hippocampus were: sampling grid size: 3 × 1 mm, counting frame size: 0.3 × 0.3 mm. The number of cells was counted manually with automatic counting correlations where we detected 3–5% of difference. All counts are expressed as the total number of cells per mm^2^.

### 4.8. Statistical Analysis

Data obtained from image analysis of the brain sections were analysed using GraphPad Prism software, version 6.01 for Windows (La Jolla, CA, USA). Data are expressed as the mean ± SD. The significance of group mean differences was evaluated by unpaired *t*-test. Behavioural analysis was performed by one-way analysis of variance (ANOVA), followed by a Student-Newman-Keuls test to compare the means of control (C) and Met-C; *p* < 0.05 was considered to be statistically significant.

## 5. Conclusions

Increased prevalence of hHcy in Western population and its role in the pathogenesis of neurodegenerative disorders makes this pathology an interesting target for future research. Our study establishes an active role of mild hHcy induced by Met enriched diet in alteration of plasma metabolome, histo-morphological alterations of CA1 hippocampal region as well as the cognitive and behavioural modifications. Our results bring insight on how the 2 g/kg Met diet can lead to the disproportion in the Met-Hcy cycle and are manifested by changes of plasma metabolome, the histo-morphological and behavioural disturbances in rats.

Apparently, prevention of the risk factors, participating in the Met-Hcy cycle might have an important prophylactic implication for the neurodegenerative disorders and deserve further investigation.

## Figures and Tables

**Table 1:** Relative changes in plasma metabolites in rats with Met diet evoked by hHcy (Met-C) against controls.

Metabolite	*p*	% Change
Glucose	*p* < 0.05	21
Acetate	*p* < 0.05	20
Lipoproteins	*p* < 0.05	−59
3-hydroxybutyrate	*p* < 0.05	53
Phenylalanine	*p* < 0.05	−17
Tryptophan	*p* < 0.05	−39
Tyrosine	*p* < 0.05	−23
Histidine	*p* < 0.05	−11
Leucine	*p* < 0.08	−14
Isoleucine	*p* < 0.08	−11
Valine	*p* < 0.08	−14

*p* derived from Mann-U-Whitney test.

## Abbreviations

CA1	Cornu ammonis 1
Hcy	Homocysteine
hHcy	Hyperhomocysteinemia
Met	Methionine
TUNEL	Terminal deoxynucleotidyl transferase dUTP nick end labeling
NMR	Nuclear magnetic resonance
